# Exploring the landscape of lymphatic filariasis research in India: A scientometric analysis of two decades from 2000 to 2024

**DOI:** 10.1371/journal.pntd.0013908

**Published:** 2026-01-06

**Authors:** Muhammed Jabir, Kannan Thiruvengadam, Pramukha Sreedevi Prabhakaran, Melissa Shaelyn Samuel, Manju Rahi

**Affiliations:** 1 ICMR-Vector Control Research Centre, Puducherry, India; 2 Saveetha Medical College and Hospital, Chennai, India; Washington University in St Louis School of Medicine, UNITED STATES OF AMERICA

## Abstract

**Background:**

The launch of the Global Programme to Eliminate Lymphatic Filariasis (GPELF) in 2000 catalysed a surge in research and programmatic initiatives worldwide. However, a systematic evaluation of global LF research output over the past two decades remains limited. This scientometric analysis maps LF research from 2000 to 2024, with a special focus on India’s contribution.

**Methods and results:**

Original research articles on LF published between 2000 and 2024 were retrieved from the Web of Science Core Collection using title-specific MeSH keywords. Bibliographic data were analysed using the *bibliometrix* package in R. A total of 1,746 documents were identified, with an average annual growth rate of 1.13%. Global output peaked in 2014, while India’s highest was in 2012. India showed a declining trend, reaching a low of 12 articles in 2018. The USA led in overall publication output (21.1%), followed by India (18.3%) and the UK (10.3%). Despite high volume, India exhibits fewer international collaborations and a moderate citation impact. Major contributors included the Indian Council of Medical Research (219 publications) and the Liverpool School of Tropical Medicine (152). Although parasitology remained a dominant theme, there was a gradual shift toward microbiology, pharmacology, and science and technology. PLOS Neglected Tropical Diseases was the most productive journal. Globally, the Gates Foundation and the National Institutes of Health were leading funders, while the Council for Scientific and Industrial Research and the ICMR were India’s leading domestic funders.

**Conclusions:**

LF research in India has shown a noticeable decline since 2017, despite its high disease burden. High-income countries dominate in citation impact and collaborations, whereas low- and middle-income countries, including India, lag behind even with a high publication volume. Strategic efforts to strengthen international partnerships, research funding, equitable co-authorship between endemic and non-endemic countries, facilitating early-career exchange programmes, adopting open-access agreements and building institutional capacity are essential to enhance the impact of LF research in India and other endemic countries. Equally important is ensuring that research priorities and implementation strategies are tailored to local programmatic and epidemiological contexts.

## Author summary

Lymphatic filariasis is a mosquito-borne disease targeted for global elimination. Since the launch of the Global Programme to Eliminate LF in 2000, scientific interest and programmatic efforts have expanded. However, how LF research has evolved during this period has not been systematically studied. We examined research articles on LF published between 2000 and 2024, focusing on global trends and India’s role. Our findings show that an initial rise in research output, followed by a decline in recent years, especially in India. High-income countries such as the USA and the UK lead influential research, often in collaboration with others. In contrast, many low- and middle-income countries tend to publish more in isolation and receive fewer citations. Recent years have seen diversification in LF research themes to areas including microbiology, pharmacology and science & technology. As the global program aims to eliminate the disease in 58 of the 72 affected countries by 2030, stronger support, collaboration, and investment are needed for endemic countries like India to sustain progress and close the final mile.

## Introduction

Lymphatic filariasis (LF) is a vector-borne neglected tropical disease (NTD) caused by *Wuchereria bancrofti, Brugia malayi, and Brugia. Timori* [1,2]. It continues to impose a significant public health burden in tropical and subtropical regions, leading to physical deformities and considerable social stigma [3,4]. Despite ongoing efforts to eliminate LF as a public health problem, approximately 657 million people in 39 countries remain at risk, with India alone accounting for ~62% of the global at-risk population [5,6]. Globally, at least 36 million people are living with the chronic manifestation of the disease [7]. In India, 2023 data reported 6,19,000 cases of lymphedema and 1,50,708 cases of hydrocele [8].

The year 2000 marked a critical turning point in the global fight against LF with the launch of the Global Programme to Eliminate Lymphatic Filariasis (GPELF) by the World Health Organisation (WHO). This coordinated international effort aimed to eliminate LF as a public health problem (EPHP) by 2030 [9]. This initiative triggered a period of intensified programmatic action and investment in preventive chemotherapy through mass drug administration (MDA), research and development of diagnostic tools and new drugs, morbidity management strategies and health systems strengthening. Since its launch, notable progress has been made globally, including the validation of 21 countries for the elimination of LF as a public health problem [10,11].

Over the past two decades, the scope of LF-related research has been expanded across several key areas, including the optimisation of elimination strategies such as MDA, two drugs (diethylcarbamazine + albendazole) and then three drugs regimen (ivermectin + diethylcarbamazine + albendazole), innovation in diagnostic technologies, vector control methods, disease modelling, and socio-behavioural studies [12–21]. It is acknowledged that research to enhance our understanding of the multiple facets of LF, develop, test and deploy new tools, assess their effectiveness against LF and also the social behavioural acceptance of the communities is going to be the driving force behind the success of the strategies and tools implemented towards ELF. However, there remains a lack of comprehensive understanding on how LF research has evolved since the inception of GPELF. This knowledge gap is especially critical amid declining global attention to NTDs, shifting donor priorities, and competing public health priorities post-COVID-19 pandemic.

Bibliometric analysis, a widely adopted method for assessing research output, has proven useful in identifying emerging patterns in research across disciplines, including public health [22]. Although one bibliometric study previously mapped LF-related research in India from 1973 to 2013, no study has systematically analysed research output during the critical years following the launch of GPELF [23]. The present study aims to address this gap by reviewing the landscape of LF-related research from 2000 to 2024, with a focus on India’s contribution. Through this 25-year analysis, we assess the volume, thematic focus, research trends, major publication outlets, funding patterns, institutions involved and collaboration in LF research. While the study does not assess the impact of these publications on LF control or policy, it provides a comparative analysis of research output between countries and regions. The findings highlight current gaps and opportunities to guide future investigations and for revitalising LF research efforts in the final stages of its elimination.

## Methods

**Fig 1:**
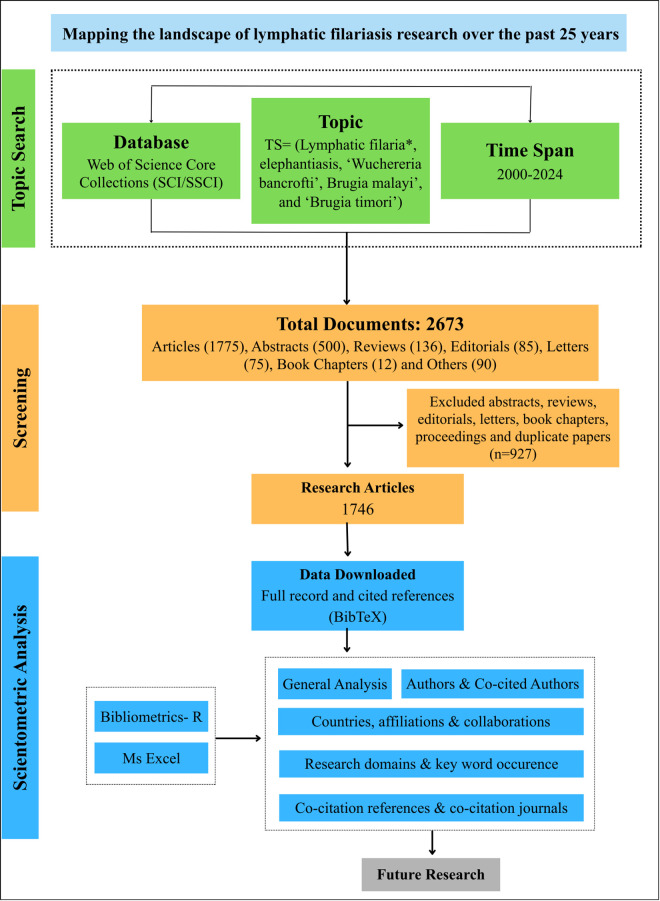
Flow diagram showing the steps of the scientometric study.

This scientometric analysis was conducted using data retrieved from the Web of Science (WoS) Core Collection, a widely recognised and trusted science citation database. The search was performed in March 2025 using the Advanced Search mode to retrieve only original research articles published between January 2000 and December 2024. The search was carried out using the following Medical Subject Headings (MeSH)-based keywords in the Title (TI) field: “Lymphatic filaria*” OR *“elephantiasis”* OR *“Wuchereria bancrofti”* OR *“Brugia malayi”* OR *“Brugia timori”.* The search was further refined with country affiliation filters using CU = (“India”) and restricted to document type “Article” to ensure the inclusion of the most relevant and peer-reviewed scholarly contributions (Fig 1). We restricted the search to “Title” rather than the broader “Topic” field to ensure that articles included had LF as the core focus of study, thereby excluding papers that merely mentioned LF peripherally. Additionally, we limited it to “Article” as the document type to ensure the inclusion of relevant and peer-reviewed research contributions. The search initially captured global LF research trends, which were subsequently refined to publications affiliated with Indian authors or institutions.

All records were exported in BibTeX and plain text formats. The metadata extracted included title, authors, year of publication, journal, institutional affiliation, author keywords, funding agencies, citation count and co-authorship data. The bibliographic data were imported into R software (version 4.5.0) and processed using the ‘bibliometrix’ package [24,25]. The bibliometric indicators analysed include publication trends over time, international and institutional collaborations patterns, top authors, institutions and journals, research domains based on WoS categories, keyword occurrence and funding agencies.

In this analysis, the country and institutional attribution of each publication were determined based on the corresponding author’s affiliation. Countries were subsequently ranked primarily according to the total number of publications, applying a full counting approach for corresponding author affiliations. Additional bibliometric indicators such as total citations (TC), average citations per publication (ACPP), single-country publications (SCP) and multi-country publications (MCP) were also considered to reflect the extent of research impact and levels of international collaboration.

Co-authorship and country-level collaboration networks were further analysed to examine the structure and strength of research partnerships in LF-related studies. The collaboration data were visualised using standard network metrics including Degree Centrality (the number of direct connections an author or country has, indicating extent of collaboration), Closeness Centrality (how close a node is to all others in the network, reflecting its efficiency in disseminating information across the network), Betweenness Centrality (how often a node acts as a bridge on the shortest path between other nodes, highlights influence and control in collaboration networks). As this study utilised only secondary, publicly available bibliographic data, no ethical approval was required.

## Results

### Global publication trends in lymphatic filariasis

**Fig 2:**
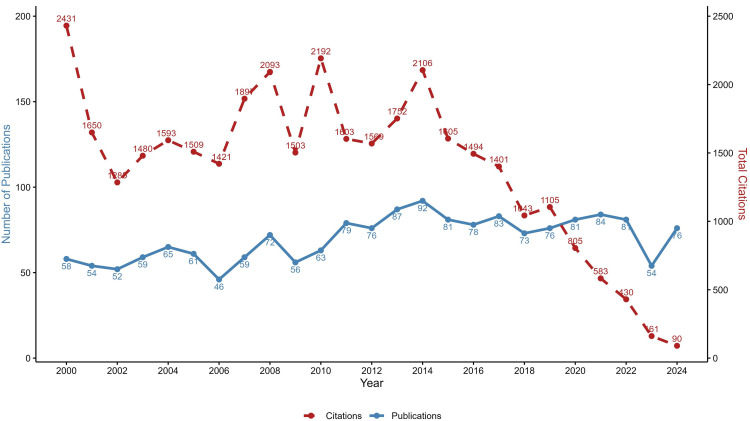
Number of LF-related publications and citations worldwide by year (2000-2024).

The search query identified a total of 1746 documents related to LF research published between 2000–2024. The overall trend reveals a fluctuating yet generally increasing line in the number of research outputs with an average annual growth rate of 1.13% (Fig 2). In the early years (2000–2006), the number of publications fluctuated, ranging from a high of 65 in 2004 to a low of 46 articles in 2006. There was a noticeable increase in the research productivity during the period from 2011 to 2021 (ranging between 73 and 87 articles annually), peaking at 92 in 2014. This increase may correspond to intensified international efforts to meet the initial GPELF target of LF elimination by 2020. In recent years (2022–2024), a decline has been observed compared to the earlier period, with only 54 publications in 2023, likely due to the impact of the COVID-19 pandemic, which disrupted the global health system and shifted research priorities. However, the numbers rebounded in 2024, rising to 76 publications, suggesting a renewed momentum in LF-related research, possibly in response to the updated WHO NTD roadmap and global target of LF elimination as a public health problem by 2030.

### Analysis by countries

**Fig 3:**
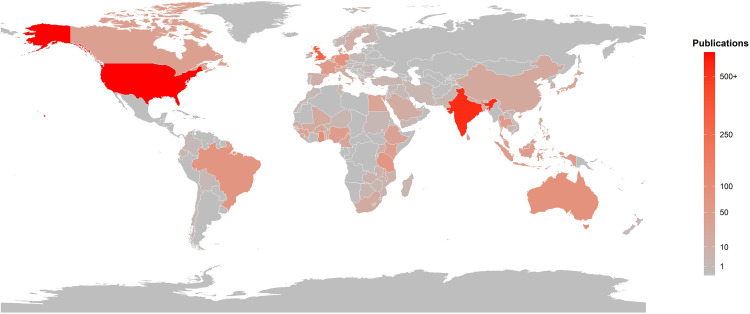
Country-wise publications on LF over 25 years based on author affiliation. The map was created in R software using base map data from Natural Earth (https://www.naturalearthdata.com). The colour gradient represents the number of publications, with darker shades indicating higher research productivity.

Table 1 provides the publication profile of the top 20 most productive countries in LF research globally. The United States of America (USA), India and the United Kingdom (UK) are the three leading contributors, cumulatively accounting for nearly half (49.7%) of all LF-related publications between 2000 and 2024 (Fig 3). The USA ranks first with 627 publications and 15671 citations, with an average citation per paper (ACPP) of 25. It has the highest Multiple Country Publication (MCP = 399), indicating strong influence in shaping the global LF research agenda, likely supported by better resources, advanced academic infrastructure and well-established global partnerships.

**Table 1:** LF-related publications, citations, SCP, and MCP of the top 20 countries (2000–2024).

SL No	Country	No. of publication	TC	ACPP	SCP	MCP
1	USA	627	15671	25	228	399
2	INDIA	542	8765	16.2	402	140
3	UNITED KINGDOM	304	10446	34.4	49	255
4	GHANA	94	2520	26.8	13	81
5	AUSTRALIA	82	1713	20.9	16	66
6	GERMANY	81	3250	40.1	8	73
7	BRAZIL	68	1056	15.5	50	18
8	TANZANIA	67	1695	25.3	8	59
9	SWITZERLAND	66	1576	23.9	5	61
10	THAILAND	54	609	11.3	37	17
11	FRANCE	51	893	17.5	14	37
12	DENMARK	50	1171	23.4	4	46
13	INDONESIA	48	1447	30.1	7	41
14	NIGERIA	48	738	15.4	11	37
15	NETHERLANDS	47	998	21.2	3	44
16	KENYA	40	915	22.9	6	34
17	CANADA	39	1425	36.5	10	29
18	SRI LANKA	34	744	21.8	13	21
19	ETHIOPIA	33	616	18.6	7	26
20	GUINEA	32	869	27.1	1	31

*TC: Total Citation.

*ACPP: Average Citation Per Paper.

*SCP: Single Country Publication.

*MCP: Multiple Country Publication.

India ranks second with 542 publications and 8765 citations, underscoring its importance as both a high-burden country and a major contributor. However, India’s relatively low MCP (140) and ACPP (16.2) suggest that a significant portion of its research is domestically focused, with limited international collaboration and relatively lower visibility. The UK, though third in terms of publication volume, nearly half of India and the US (304 publications and 10446 citations), outperforms others in terms of impact, with an ACPP of 34.4, indicating higher quality and widely cited research. Countries such as Germany, with fewer publications (81), also demonstrate strong academic influence (ACPP = 40.1) and substantial international collaboration (MCP = 73), striking a balance between quality and global engagement. Several low- and middle-income endemic countries, including Ghana, Nigeria, Ethiopia, and Tanzania, contribute meaningfully to LF research. Ghana, in particular, stands out with a high ACPP of 26.8, suggesting impactful research, possibly due to international collaborations.

**Fig 4:**
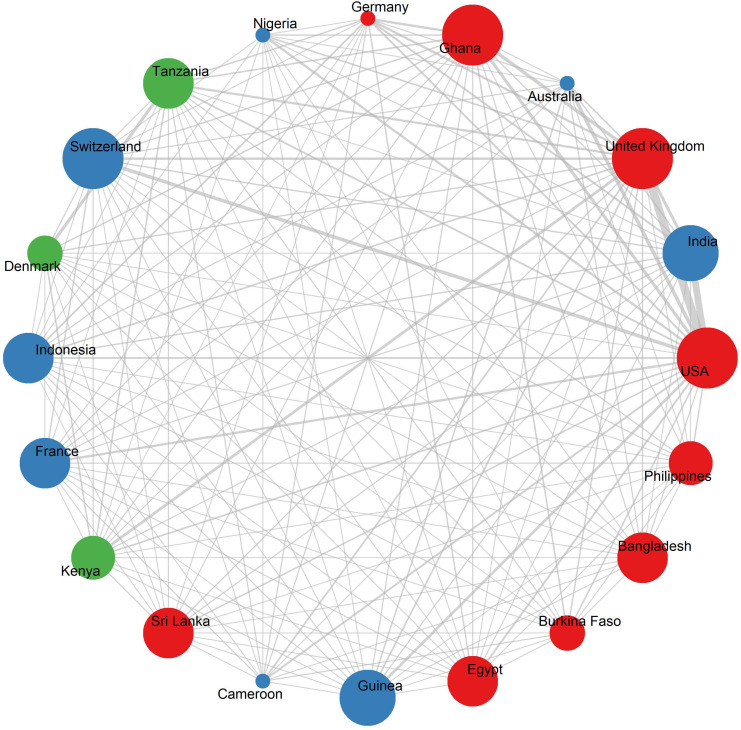
Network visualisation map of international research collaboration in LF research. The node size is proportional to publication output and collaboration frequency, while edge thickness reflects the strength of bilateral connections. The map shows three distinct clusters of collaboration. The red cluster, including the USA and UK, represents global leaders in collaboration with widespread partnerships. The blue cluster comprises countries from Asia and Oceania, such as India and Australia, highlighting strong regional ties. The green cluster includes sub-Saharan African and some European countries, such as Ghana, Tanzania, Switzerland, and France, showing focused collaborations in those regions.

The country collaboration network illustrates how nations around the world work together in LF research through international partnerships (Fig 4). To understand the structure of these research partnerships, co-authorship collaboration networks were analysed using standard network analysis metrics: degree centrality, closeness centrality, and betweenness centrality. *Degree centrality* indicates the level of collaboration a country has, *closeness centrality* reflects how efficiently it can access and disseminate information across the network, and *betweenness centrality* captures its role as a connector or bridge within the network.

The US emerged as the most connected country in the network, demonstrating the highest degree of collaboration (degree centrality = 62), strong integration with other countries (closeness = 0.79), and a key bridging role between different groups (betweenness = 0.18). It also leads in collaborative research output (1359 publications), and holds the highest citations (41990). The UK ranks second across all three metrics, with strong connections (degree = 56), good network reach (closeness = 0.75), and a bridging role (betweenness = 0.13), reflecting its extensive collaborative footprint. Among endemic countries, India and Ghana demonstrate strong regional and international partnerships. India ranks just behind the USA in collaborative publications (1047) and degree of collaboration (40). However, its moderate betweenness value (0.071) indicates that there is still room to increase its collaborative reach and leadership in bridging global LF research efforts. Ghana, with a degree of 44 and a betweenness of 0.0307, plays a significant role in sub-Saharan Africa, reflecting its growing leadership in regional LF research networks. Countries such as Tanzania, Indonesia, Sri Lanka, and Switzerland also exhibit a moderate degree of collaboration (ranging from 33 to 39), suggesting their active engagement in LF research efforts.

### Analysis by institutions

A total of 932 institutions/research bodies, either independently or in collaboration, contributed to LF research between 2000 and 2024. Among these, eight institutions made particularly significant contributions, each publishing 50 or more articles, and collectively accounting for 847 publications (48.5%). The Indian Council of Medical Research (ICMR) led with 219 publications. The Liverpool School of Tropical Medicine (LSTM) and the Council of Scientific and Industrial Research (CSIR), India, followed with 152 and 106 publications, respectively. Other major contributors include University of Liverpool (101), Washington University (91), the Centres for Disease Control and Prevention (CDC) (87), the National Institutes of Health (NIH) (80), Banaras Hindu University (71), the World Health Organisation (WHO) (66), Anna University (64) and the University System of Ohio (51).

The global institutional collaboration network shows the roles of key organisations driving LF research (S1 Fig). The LSTM ranks highest in closeness (0.513) and has one of the highest betweenness scores (0.077), indicating that it is not only well-connected with a large number of institutions but also acts as a key intermediary in collaborative chains. Although the ICMR has a slightly lower closeness score (0.487), it records the highest betweenness centrality (0.089), suggesting it plays an important bridging role, likely connecting domestic Indian research institutions with international collaborators. The University of Liverpool (closeness: 0.493; betweenness: 0.037) and Washington University (closeness: 0.490; betweenness: 0.045) also show strong central positioning in cross-institutional linkages. Similarly, the WHO and the NIH, with notable betweenness values of 0.043 and 0.055, respectively, are positioned as globally influential actors that facilitate multilateral coordination across research institutions and knowledge translation.

### Analysis by domain and keywords

**Fig 5:**
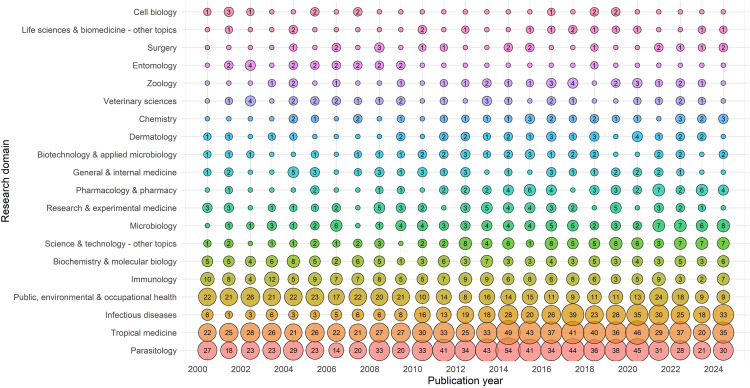
Research domains with the most publications on LF over 25 years. Each bubble represents the number of publications in a given year. Larger bubbles indicate a higher number of publications and topics dominated in different years.

A domain-wise trend analysis of LF-related publications from 2000 to 2024 is presented in bubble plots (Fig 5). Parasitology, Tropical Medicine, and Infectious Diseases have consistently been the leading subject categories in LF research over the past decades. Parasitology remains the most dominant domain, with 783 publications, reflecting the continued focus of researchers on the biology and lifecycle of LF-causing parasites such as *Wuchereria bancrofti*, *Brugia malayi,* and *Brugia timori.* It maintained a strong presence throughout the study period and recorded high publication counts even in recent years (e.g., 45 in 2020), indicating sustained research interest. Closely following is ‘Tropical Medicine’ with 779 publications, highlighting the public health orientation of LF as a tropical disease. This domain also showed robust outputs across years, peaking in 2020 with 46 publications.

Though slightly behind the top two, ‘infectious disease’ has shown a steady increase, particularly after 2010, with a notable peak of 39 publications in 2017. This suggests a growing interdisciplinary engagement with LF, including co-infection studies and integration into broader infectious disease frameworks. After 2010, LF research began diversifying into other domains such as Microbiology, Science & Technology, and Pharmacology, likely influenced by advancements in technology, diagnostics, and drug development. This evolving disciplinary landscape is essential for supporting integrated elimination strategies, innovations in treatment and diagnostics and improved vector control measures. Between 2000 and 2024, the most frequently used keywords by authors are *lymphatic filariasis* (226 mentions), *infection* (219), and *Wuchereria bancrofti* (202). Keywords associated with the mass drug administration (MDA) strategy, such as *diethylcarbamazine* (177), *ivermectin* (159), and *albendazole* (132) also used prominently, indicating sustained interest in drug-based elimination efforts. These drug-related terms showed notable peaks between 2013 and 2016, likely due to the global scale-up of MDA and the introduction of alternative drug regimens such as triple-drug therapy (IDA) (S2 Fig).

### Top journals publishing LF-related research globally

The top ten most productive journals, each publishing ≥50 articles, collectively account for 41.3% of all LF-related publications during the assessment period (S3 Fig). *PLOS Neglected Tropical Diseases* (PNTD) emerged as the leading outlet, publishing 238 articles. Other notable journals include the *American Journal of Tropical Medicine and Hygiene* (AJTMH) (112 publications), *Parasites & Vectors* (79), *Transactions of the Royal Society of Tropical Medicine and Hygiene* (TRSTMH) (73), *Parasitology research* (61), *Acta Tropica* (56), *Annals of Tropical Medicine and Parasitology* (53), *Tropical Medicine and International Health* (50) *PLOS One* (48) and *Molecular and Biochemical Parasitology* (42).

### Research domains and journals

**Fig 6:**
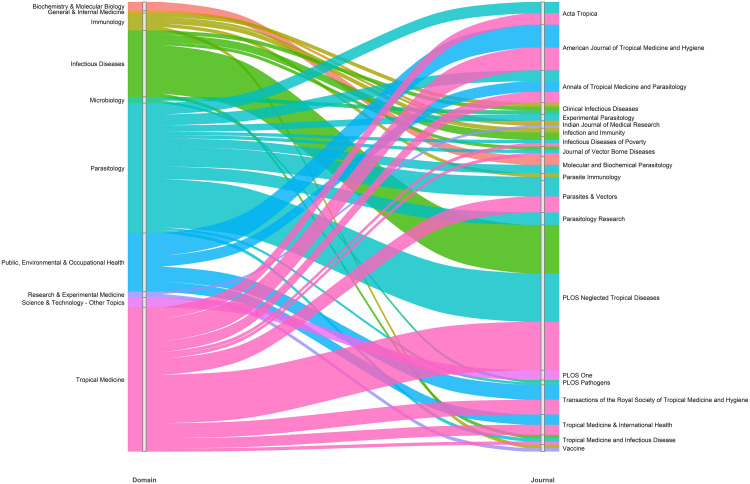
Top research domains and journals in LF research over 25 years. Each coloured flow represents the link between the research domain and the journals in which related articles were published. The thickness of each flow corresponds to the relative number of publications.

The alluvial plot (Fig 6) shows the links between the top ten research domains and their corresponding journals publishing LF research. The strongest connections are seen between the leading domains, such as ‘Infectious Diseases’, ‘Parasitology’, and ‘Tropical Medicine’ and major journals such as *PNTD* and the *AJTMH*. *PNTD* is the most influential journal, receiving substantial contributions from all three leading domains, each accounting for 13.6% of total articles. *AJTMH* also draws significant research from the ‘Public Health’ and ‘Tropical Medicine’, each contributing 6.4% of total publications. Beyond these, the journals such as *Parasites & Vectors, Parasite Immunology*, and the *TRSTMH* are key publication outlets connecting to ‘Parasitology’ and ‘Tropical Medicine’ domains. Additionally, journals like *Acta Tropica* and *Annals of Tropical Medicine* and *Parasitology* receive multidisciplinary research, reflecting cross-domain interest in LF biology, public health, and experimental medicine.

### Funding agencies

**Fig 7:**
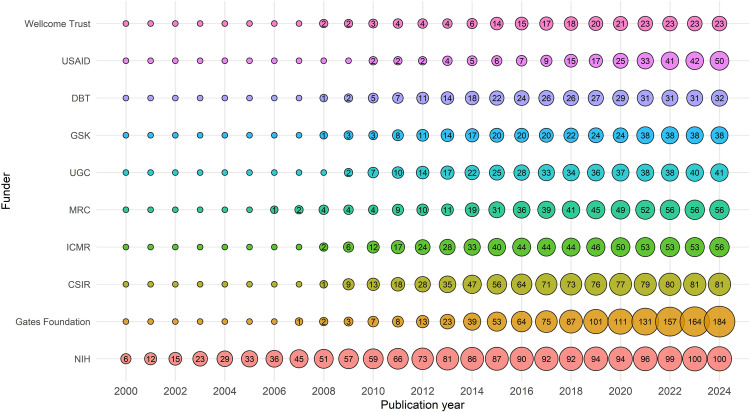
Evolution of the top 10 funding agencies in LF research over 25 years. Each bubble represents the number of publications supported by the funding agencies in a given year, and the size of the bubble corresponds to the publication volume.

Fig 7 presents the annual cumulative funding contribution from the world’s top 10 agencies supporting LF research. Over the last two decades, research on LF has been supported by a diverse group of national and international funding agencies. A total of 93 funding agencies supported LF-research globally during the period 2000–2024. The trend analysis shows that the funding has been primarily driven by both philanthropic foundations and government-based agencies. Among the top 15 funders, the Gates Foundation (GF) and the NIH were the two most prominent, each supporting over 160 grants. NIH support has remained steady since the early 2000s, reflecting its long-term commitment to LF research, while GF’s involvement became notably visible after 2013 and has grown rapidly since then. Indian research agencies such as the CSIR and the ICMR have also made significant contributions, each funding more than 70 projects. Other key contributors include the Medical Research Council (MRC, UK) (74 grants) and GlaxoSmithKline (GSK) (39 grants), illustrating the strength of public-private collaboration. The Wellcome Trust, WHO and the United States Agency for International Development (USAID) have also made meaningful contributions to advancing LF research globally.

### LF-related publications in India: volume, types and growth

**Fig 8:**
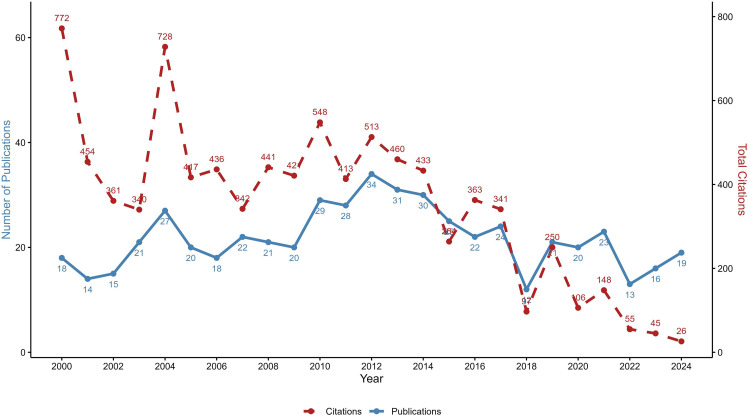
LF-related publications and citations in India over 25 years.

India’s contribution to LF-related publications has been significant, though with notable fluctuations over the past two decades. A total of 542 publications were identified during the study period. The early 2000s saw a steady rise in the number of publications, peaking in 2012 with 34 publications. This period also recorded relatively high citation counts, with 2004 standing out as the most cited year (728 citations), reflecting seminal works published in those years. However, from 2015 onwards, there has been a gradual decline in both publications and citations. In 2018, the output dropped its lowest, with just 12 articles and 97 citations, possibly due to shifts in research priorities, funding limitations, or external challenges. In recent years (2020–2024), although publication numbers remain moderate (13–23 papers annually), citation counts declined sharply, especially in 2023 (45 citations) and 2024 (26 citations). This downward trend suggests the need for renewed investment and strategic focus in LF research to sustain momentum toward elimination goals (Fig 8).

### Top contributing institutes in LF research in India

Table 2 presents the top five leading institutions/research bodies contributing to LF research in India based on publication output and citation counts. The ICMR stands out as the top contributor, with 219 publications and 4277 citations. The ICMR is the nodal research body in India under the Ministry of Health and Family Welfare (MoHFW), responsible for the formulation, coordination and promotion of biomedical research. It has 28 permanent institutes across the country, each with a mandate to address specific disease or regional health issues. Among these, the Vector Control Research Centre (VCRC), based in Puducherry, focuses on vector-borne diseases, and serves as a WHO collaborating centre for research and training in LF. VCRC ranks highest among ICMR institutes in terms of LF research output, contributing 131 publications and 2,413 citations. The CSIR follows with 106 publications and 1,404 citations. Banaras Hindu University (71 publications, 952 citations), Anna University (64 publications, 1124 citations) and Mahatma Gandhi Institute of Medical Sciences, Sevagram (48 publications and 872 citations) also demonstrate strong academic engagement in LF research.

**Table 2:** Top five institutes/research bodies in LF research in India between 2000-2024.

Rank	Indian author’s affiliation	No of Publication	Total Citation
1	Indian Council of Medical Research (ICMR), New Delhi	219	4277
2	Council of Scientific and Industrial Research (CSIR), New Delhi	106	1404
3	Banaras Hindu University, Uttar Pradesh	71	952
4	Anna University, Tamil Nadu	64	1124
5	Mahatma Gandhi Institute of Medical Sciences, Maharashtra	48	872

### Top journals publishing LF-related research in India

The top 10 most productive journals, each publishing more than 10 articles, contributed 208 papers, accounting for 38.3% of the cumulative publication output. *Parasitology Research* leads with 32 papers and 473 citations, followed closely by *PLOS Neglected Tropical Diseases*, which has 31 publications and the highest citation count of 597, reflecting its strong academic impact. Other notable journals include *Acta Tropica* (27 papers), *Annals of Tropical Medicine and Parasitology* (24), and *Transactions of the Royal Society of Tropical Medicine and Hygiene* (20). Indian journals such as the *Journal of Vector-Borne Diseases* (15) also play a significant role in LF research dissemination. Among these journals, *Vaccine* stands out with the highest impact factor of 5.2 (S4 Fig).

### India and international research collaborations

The international co-authorship network of Indian authors in LF-related publications highlights the USA (degree = 30; closeness 0.8) and the UK (degree = 26; closeness = 0.74) as India’s strongest international partners. Other active partners include Kenya and Sri Lanka (each with a degree of 20), followed by Guinea (18), Switzerland (17), Ghana (17), Tanzania (16) and Germany (15) (S5 Fig).

### Top terms trends in India

The domain-wise analysis of LF-related publications in India from 2000 to 2024 reveals a strong interdisciplinary research focus. ‘Parasitology’ remains the most dominant field, with 218 publications. Tropical Medicine ranks second, especially active between 2000 and 2015, with 188 publications. From 2015 onward, ‘Biochemistry and Molecular Biology’ shows more frequent entries. Emerging interest in ‘Chemistry and Biotechnology’ in recent years signals a gradual shift toward innovation in diagnostics and therapeutics. Notably, Chemistry, ‘Biotechnology & Applied Microbiology’, and ‘Pharmacology & Pharmacy’ have emerged as important research domains in recent years. This shift signifies a gradual movement toward innovation in diagnostics, drug development, and therapeutic interventions (S6 Fig).

### Citation analysis

The 543 papers reviewed have collectively received 8,774 citations. Table 3 presents the top 10 most cited articles on LF in India during the study period. The most cited publication by Ramaiah et al. (2000), in *Parasitology Today*, received 192 citations and examined the economic burden of LF in India. Other frequently cited works addressed key areas such as immune-regulatory disruptions by filarial parasites (Babu, Subash, et al., 2006; 179 citations), genetic perspective on susceptibility to *W. bancrofti (*Choi, et al.; 2001; 115 citations)*,* impairment of tetanus-specific immune responses in individuals with LF (Nookala, Suba, et al.; 2004; 105 citations) and operational challenges in mass drug administration (Babu, B. V., and Sathyanarayana K. Kar, 2004; 102 citations).

**Table 3:** Top 10 highly cited articles in India.

*Rank*	*Authors*	*Title*	*Source Title*	*Year*	*Citation*
1	Ramaiah, K. D., et al.	The economic burden of lymphatic filariasis in India	Parasitology today	2000	192
2	Babu, Subash, et al.	Regulatory networks induced by live parasites impair both Th1 and Th2 pathways in patent lymphatic filariasis: implications for parasite persistence.	The journal of immunology	2006	179
3	Choi, E. H., et al.	Genetic polymorphisms in molecules of innate immunity and susceptibility to infection with Wuchereria bancrofti in South India.	Genes & Immunity	2001	115
4	Nookala, Suba, et al.	Impairment of tetanus-specific cellular and humoral responses following tetanus vaccination in human lymphatic filariasis	Infection and immunity	2004	105
5	Babu, B. V., and Sathyanarayana K. Kar.	Coverage, compliance and some operational issues of mass drug administration during the programme to eliminate lymphatic filariasis in Orissa, India.	Tropical Medicine & International Health	2004	102
6	Babu, Subash, et al.	Diminished expression and function of TLR in lymphatic filariasis: a novel mechanism of immune dysregulation	The Journal of Immunology	2005	100
7	Ramaiah, K. D., et al.	A programme to eliminate lymphatic filariasis in Tamil Nadu state, India: compliance with annual single-dose DEC mass treatment and some related operational aspects.	Tropical Medicine & International Health	2000	94
8	Gnanasekar, Munirathinam, et al.	Novel phage display-based subtractive screening to identify vaccine candidates of Brugia malayi.	Infection and immunity	2004	90
9	Nutman, Thomas B., and V. Kumaraswami.	Regulation of the immune response in lymphatic filariasis: perspectives on acute and chronic infection with Wuchereria bancrofti in South India.	Parasite immunology	2001	89
10	Norman, R. A., et al.	EPIFIL: the development of an age-structured model for describing the transmission dynamics and control of lymphatic filariasis.	Epidemiology & Infection	2000	87

### Top funding agencies in India

**Fig 9:**
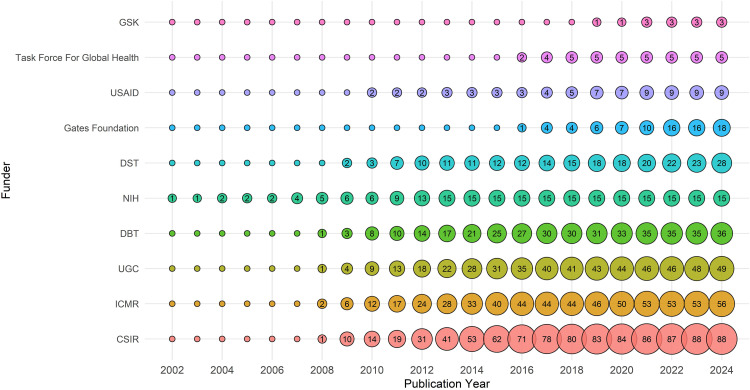
Top funding agencies in India for LF research over 25 years. The number in the bubbles indicates the number of publications supported by the funding agencies in a given year.

The funding landscape for LF research in India reflects strong support from both national and international agencies. Among the top 15 funders, the CSIR and the ICMR are the two most consistent domestic contributors, providing 79 and 70 grants, respectively (Fig 9). Among international agencies, the GF and the NIH are equally prominent, each supporting 23 projects, highlighting significant global philanthropic and scientific engagement. Other major national funders include the DBT with 25 projects and the University Grants Commission (UGC) with 7 projects. This diverse funding ecosystem highlights the collaborative nature of LF research in India, with strong governmental backing and complemented by global partnerships. However, sustained funding from both domestic and global agencies will be essential to maintain research momentum.

## Discussion

This scientometric analysis provides a comprehensive overview of global and Indian research trends in LF from 2000 to 2024, highlighting publication outputs, collaborative networks, thematic shifts, and funding patterns. The publication trajectory reveals substantial fluctuations over time. There was a noticeable increase in research productivity during the period from 2011 to 2017. Global output peaked in 2014 with 92 publications, and Indian output in 2012 (34 publications), likely attributed to the momentum generated toward achieving the 2020 GPELF elimination target. However, a subsequent decline, particularly in India with the lowest output in 2018 (12 publications), raises concerns about waning research attention. While global research shows a modest recovery in 2024, India’s continuing downward trend is concerning and highlights the need for renewed focus, investment, and capacity building in LF research.

Geographically, LF research is concentrated in a few high-income or high-burden countries, with the USA (21.1%) and India (18.3%) leading in publication volume. However, India’s comparatively low MCP ratio and moderate citation impact point to limited engagement in international research networks. Previous literature has highlighted the poor research output from India’s medical institutions [26]. In contrast, the USA and European nations such as the UK, Germany and the Netherlands exhibit higher MCP ratios and citation metrics, suggesting their strategic positions in transnational research networks. These findings align with broader patterns in global health research, which often reflect disparities between high-income and low- and middle-income countries in research leadership [27–29]. To bridge these disparities, there is a clear need to strengthen both South–South and South–North research partnerships. Encouraging equitable co-authorship between endemic and non-endemic countries, facilitating early-career researcher exchange programmes, and adopting transformative open-access agreements could accelerate capacity building and knowledge sharing. Such mechanisms can ensure that research agendas better reflect local priorities and that endemic regions are more represented in the global research agenda.

Overall, the high-income countries continue to dominate in terms of citation impact. Although the middle-and low-income countries, including India, contribute significantly in publication numbers, they have low ACPPs, possibly due to less frequent publication in high-impact journals and limited international collaborations. The systemic challenges, such as inadequate research funding and the high article processing charges (APCs) associated with open-access journals, may pose significant barriers. Researchers in India and other South-East Asian countries, who often lack institutional or external financial support, find it difficult to afford APCs and publish in widely read, high-impact journals. Notably, APCs have increased far beyond inflation over the past two decades, with median total paid by high-impact researchers being USD 2900 in 2019 [30]. Scientists from low- and middle-income countries (LMICs) are particularly disadvantaged in this regard, as they often lack support to cover APCs required [31]. Although some funding agencies and universities provide APC support, such provisions are inconsistent, especially in LMIC contexts. Waiver schemes exist in certain journals to mitigate this barrier, but their eligibility criteria vary widely and are not universally accessible [32,33]. Therefore, it is important to evaluate the financial burden of publishing on researchers in resource-limited settings and establish mechanisms to promote equitable participation in global scientific communication.

At the institutional level, the ICMR, LSTM, and US agencies, including NIH and CDC, are central to the global LF research ecosystem. The ICMR and LSTM, with high betweenness and closeness centrality scores, act as bridging knowledge hubs within their respective regional and global networks. The prominence of ICMR illustrates the strength of local research institutions in addressing context-specific LF challenges and plays a key role in shaping the healthcare landscape in India [34].

Thematic analysis reveals the dominance of traditional research domains such as parasitology, tropical medicine, and infectious diseases. However, since 2015, a noticeable diversification has occurred, with growing interest towards microbiology, pharmacology, biotechnology, and applied sciences. This trend reflects emerging research priorities around diagnostics, drug development and other areas [35]. The surge in keywords such as ‘ivermectin’, ‘albendazole’, and ‘diethylcarbamazine’ between 2013 and 2016 correlates with the global rollout of triple-drug therapy (IDA) under the GPELF [36]. Future LF research should increasingly focus on programmatically aligned domains that are critical to achieving LF elimination goals. Greater emphasis is needed on operational and implementation research addressing MDA adherence strategies, post-validation surveillance mechanisms, scalable models of MMDP, and the integration of LF services within routine primary health care (PHC) platforms. Prioritising these research areas will ensure that scientific output is better aligned with on-ground programme needs.

The *PLOS Neglected Tropical Diseases* has been identified as the leading journal in disseminating research outputs from LF-related studies, publishing 238 articles globally and 31 from India. Funding agencies and financial grants play an important role in advancing scientific research across disciplines and areas. The study identifies 93 national and international funding bodies that supported LF research. Among them, philanthropic organisations such as the GF and public institutions like the NIH and the CDC have played instrumental roles in sustaining global LF research efforts. A 2016 study identified 55 key funders actively supporting global health research, with the NIH, European Commission, and UK Medical Research Council being the top contributors internationally. Among philanthropic funders, the Wellcome Trust was reported as the largest supporter of health research [37]. In India, government-funded scientific research bodies such as CSIR and ICMR have been pivotal, contributing 79 and 70 grants, respectively. International funders, including GF, NIH, and CDC, have also played significant roles in India, collectively supporting 50 LF-related studies, highlighting the importance of strong public-private and global partnerships.

Beyond financial contributions, funding mechanisms play an important role in determining the thematic direction of LF research. Many of the research studies appear to follow the priorities of major funders and grant calls. While their contributions are high in facilitating LF research, their priorities may not always align with locally identified needs or context-specific operational challenges. Therefore, strong leadership and governance is needed to ensure that funding is not only acquired but strategically aligned towards programme-relevant research domains. Mechanisms such as priority-setting consultations with stakeholders from endemic countries, and alignment frameworks between governments, academia, and implementing agencies can support a context-responsive research ecosystem.

This study was limited to indexed articles retrieved from the Web of Science and, therefore, may not fully represent all research conducted in LF particularly unpublished research, non-indexed articles, or grey literature. Although such sources may provide additional insights into LF research activity, their inclusion falls beyond the scope of scientometric analysis, which relies on indexable bibliometric records. Furthermore, although citation counts were used as a key indicator for research impact in this study, they may not reflect the real-world influence of research on policy formulation or programme decisions.

## Conclusion

Over the past two decades, research on LF has shown notable fluctuations, with a marked increase in publications around 2017 followed by a slight decline in 2018. The US and India remain the leading contributors in terms of research output. The most active domains of LF research include Parasitology, Tropical Medicine, and Infectious Diseases. Key institutions such as the ICMR, LSTM, NIH and CDC occupy a central position in the global LF research network. PNTD and AJTMH serve as major dissemination platforms. Research funding was largely driven by public agencies, philanthropic organisations, and academic institutions, with significant contributions from the GF, NIH, CSIR and ICMR. While global LF research has shown renewed momentum in recent years, India’s declining publication trend since 2017 is concerning, particularly given its high disease burden. Although contributing significantly, India’s comparatively lower international collaboration and moderate citation impact reflect limited integration into global research networks. In contrast, high-income countries continue to lead in research influence. Strengthening India’s position and research visibility requires greater participation in multi-country research projects, stronger institutional linkages and increased investment in research infrastructure. While Indian agencies such as ICMR and CSIR play a leading role, building robust partnerships that link Indian academia with globally recognised research institutions, public health programmes, and funding agencies is essential. Building a sustainable research ecosystem in India requires targeted capacity-building initiatives such as early-career researcher exchange programmes, and measures to reduce the financial barriers associated with high publication costs. Expanding open-access agreements and supporting Indian scholars to publish in high-impact journals can further increase India’s research visibility and citation impact. Although LF research domains has diversified since 2015, a greater emphasis is required on operational and implementation research that addresses programme- relevant priorities. As many countries, particularly India, approach the ‘last mile’, renewed investment in research and evidence generation is essential to maintain momentum and achieve successful LF elimination in the coming years.

## Supporting information

S1 Fig Collaboration among the top 20 institutes. (TIFF)

S2 Fig Top keywords. (TIFF)

S3 Fig Most productive journals. (TIFF)

S4 Fig Highly productive journals in India. (TIFF)

S5 Fig Network visualisation map of research collaboration of Indian institutions in LF. (TIFF)

S6 Fig Research domains with the most publications on LF in India over 25 years. (TIFF)
